# Can Xanthophyll-Membrane Interactions Explain Their Selective Presence in the Retina and Brain?

**DOI:** 10.3390/foods5010007

**Published:** 2016-01-12

**Authors:** Justyna Widomska, Mariusz Zareba, Witold Karol Subczynski

**Affiliations:** 1 Department of Biophysics, Medical University of Lublin, 20-090 Lublin, Poland; 2 Department of Ophthalmology, Medical College of Wisconsin, Milwaukee, WI 53226, USA; mariusz@mcw.edu; 3 Department of Biophysics, Medical College of Wisconsin, Milwaukee, WI 53226, USA; subczyn@mcw.edu

**Keywords:** carotenoids, macular xanthophylls, zeaxanthin, lutein, neural tissue, lipid antioxidants, age-related macular degeneration (AMD), age-related neurodegenerative diseases

## Abstract

Epidemiological studies demonstrate that a high dietary intake of carotenoids may offer protection against age-related macular degeneration, cancer and cardiovascular and neurodegenerative diseases. Humans cannot synthesize carotenoids and depend on their dietary intake. Major carotenoids that have been found in human plasma can be divided into two groups, carotenes (nonpolar molecules, such as β-carotene, α-carotene or lycopene) and xanthophylls (polar carotenoids that include an oxygen atom in their structure, such as lutein, zeaxanthin and β-cryptoxanthin). Only two dietary carotenoids, namely lutein and zeaxanthin (macular xanthophylls), are selectively accumulated in the human retina. A third carotenoid, *meso*-zeaxanthin, is formed directly in the human retina from lutein. Additionally, xanthophylls account for about 70% of total carotenoids in all brain regions. Some specific properties of these polar carotenoids must explain why they, among other available carotenoids, were selected during evolution to protect the retina and brain. It is also likely that the selective uptake and deposition of macular xanthophylls in the retina and brain are enhanced by specific xanthophyll-binding proteins. We hypothesize that the high membrane solubility and preferential transmembrane orientation of macular xanthophylls distinguish them from other dietary carotenoids, enhance their chemical and physical stability in retina and brain membranes and maximize their protective action in these organs. Most importantly, xanthophylls are selectively concentrated in the most vulnerable regions of lipid bilayer membranes enriched in polyunsaturated lipids. This localization is ideal if macular xanthophylls are to act as lipid-soluble antioxidants, which is the most accepted mechanism through which lutein and zeaxanthin protect neural tissue against degenerative diseases.

## 1. Introduction

**Figure 1:**
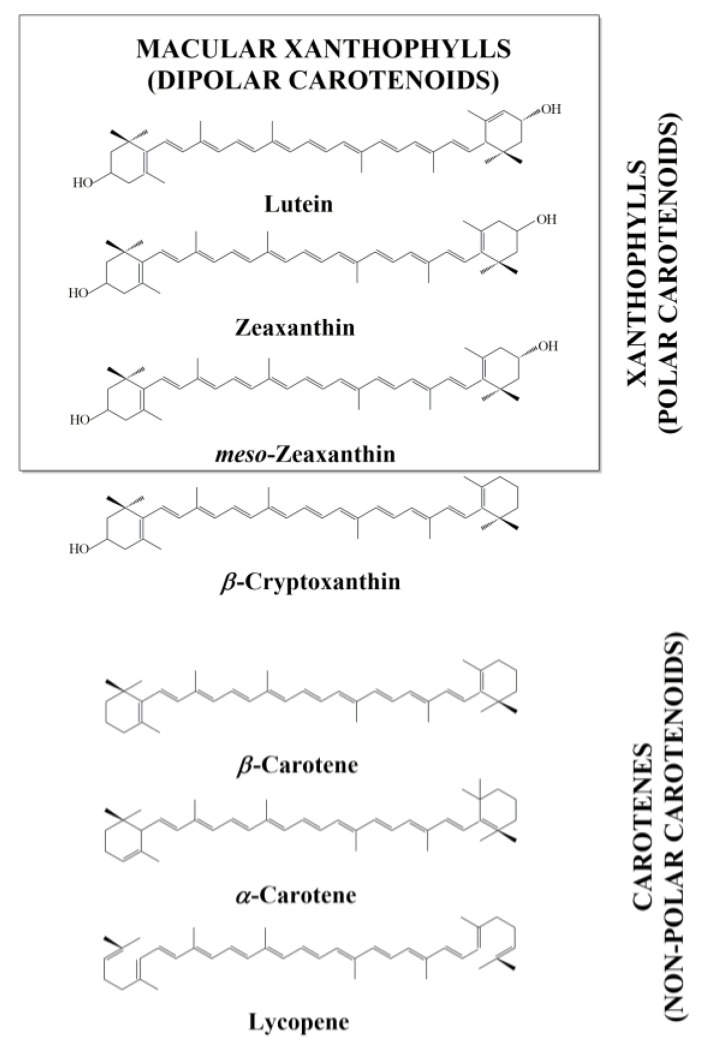
Chemical structures of carotenoids (xanthophylls and carotenes) abundant in food, blood plasma and neural tissue.

**Figure 2:**
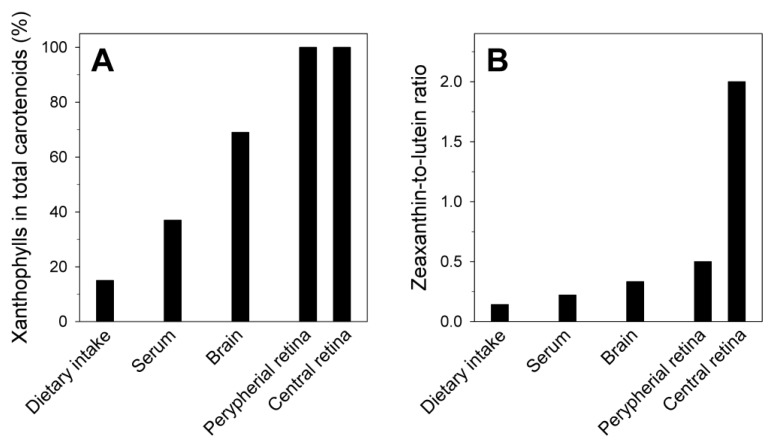
(**A**) Preferential accumulation of xanthophylls in the brain and retina tissues expressed as a percent of xanthophylls in the total carotenoid pool; (**B**) preferential accumulation of zeaxanthin over lutein in the brain and retina tissues expressed as the zeaxanthin-to-lutein ratio. Data adapted from [1,2,4,9,10,11,12].

Major carotenoids that are present in the human diet can be divided into two groups, carotenes (nonpolar carotenoids, such as β-carotene, α-carotene and lycopene) and xanthophylls (polar carotenoids, such as lutein, zeaxanthin and β-cryptoxanthin) (see Figure 1 for their structures). Xanthophylls form less than 20% of the total carotenoids in the human diet. Already in the blood plasma, the amount of xanthophylls increases to about 40% (Figure 2A) [1]. It should be noted that the serum xanthophyll-to-carotenoid ratio varies among the population and depends on diet. This preferential intake of xanthophylls is further enhanced at the level of neural tissues. Xanthophylls are preferentially accumulated from circulation into the brain [1,2] and retina [3,4]. Recently, several carotenoids (including lutein, zeaxanthin, β-cryptoxanthin, α-cryptoxanthin, β-carotene, α-carotene and lycopene) have been identified in the human brain [2]. Surprisingly, xanthophylls are the most abundant carotenoids in this tissue; the evaluation by Craft *et al.* [2] indicates that xanthophylls account for about 65% of total carotenoids in all brain regions. Similar preferential accumulation of up to 72% of xanthophylls from circulation into the brain was reported by Johnson *et al.* [1]. Thus, brain tissue, similar to the retina, preferentially accumulates macular xanthophylls (lutein and zeaxanthin); additionally, it accumulates cryptoxanthin, which is not present in the retina. This preferential accumulation is maximal in the case of the retina, where only xanthophylls are present [3,4]. Figure 2A illustrates the enhanced tissue preferences and selectivity for the accumulation of xanthophylls.

Only two dietary carotenoids, namely lutein and zeaxanthin (macular xanthophylls), are selectively accumulated in the human retina. The highest concentration of macular xanthophylls is found in the outer plexiform layer, which is a layer of neuronal synapses between photoreceptor cells and secondary neurons [3,5]. Macular xanthophylls are also present in the layer of rod outer segments [6,7] and in retinal pigment epithelium cells [8]. In addition to the preferential accumulation of xanthophylls from food into the neural tissue, there is a significant increase in the zeaxanthin-to-lutein ratio in neural tissue, as compared to that in the dietary intake of these xanthophylls, and in blood plasma. In human serum, the zeaxanthin-to-lutein ratio ranges from 1:7 to 1:4 [1,9,10,11,12], which is consistent with the relatively high lutein content in fruits and vegetables as compared to the content of zeaxanthin.

Zeaxanthin is the dominant xanthophyll in only a few food products, such as the goji berry and orange pepper [13,14]. Thus, the dietary intake of lutein is much higher than that of zeaxanthin, with the evaluated dietary zeaxanthin-to-lutein ratio of 1:12 to 1:5 [11,15,16]. As indicated in Figure 2B, this ratio is increased first on the level of the serum and next when macular xanthophylls are selectively accumulated in the neural tissue. The zeaxanthin-to-lutein ratio in the retina increases to the value of 1:2 in the retina periphery and 2:1 in the central macula [17]. The significant part of the total retina zeaxanthin is represented by its stereoisomer *meso*-zeaxanthin (see Figure 1 for its structure), which is formed directly in the retina from lutein [17]. The reported zeaxanthin-to-lutein ratio in the human brain ranges from 1:3 to 1:1.4 [1,2]. All of these data suggest that xanthophylls are selectively accumulated and stored in neural tissue and indicate that zeaxanthin (and *meso*-zeaxanthin) has a particular role in the neural tissue different from that of lutein. It is commonly accepted that both lutein and zeaxanthin are potent lipid-soluble antioxidants. However, the data presented in Figure 2B demonstrate that the retina and brain, which are tissues susceptible to lipid peroxidation, preferentially accumulate zeaxanthin from a human diet poor in zeaxanthin. Furthermore, the synthesis of *meso*-zeaxanthin in the retina directly from lutein invites the intriguing question: Is the appearance of *meso*-zeaxanthin in the retina a tissue response to a zeaxanthin-poor diet?

In plants, xanthophylls are present in the pigment-protein complexes of the photosynthetic apparatus [18]. As lipid-soluble molecules, they are also associated with the lipid-bilayer portion of the photosynthetic plant apparatus [18,19,20], especially under light stress conditions [21]. Their presence in the lipid-bilayer portion of membranes in prokaryotes is well established [22,23,24]. In these organisms, xanthophylls are associated with membranes subjected to light and oxygen stress. These membranes are particularly rich in polyunsaturated lipids and, under oxidative stress, produce high levels of reactive oxygen species (ROS). These two features of plant thylakoid and prokaryote membranes are shared with the retina photoreceptor segment and neuron membranes. The light harvesting system of plants contains about 75% polyunsaturated lipids [25]. Similarly, photoreceptors of the retina and the neuronal endings and synaptosomes are very rich in long-chain polyunsaturated fatty acids (PUFAs), especially docosahexaenoic acid (DHA) [26,27,28]. In all of these tissues, only polar carotenoids, namely xanthophylls, are present as lipid-soluble antioxidants. Functional association of xanthophylls as a lipid-soluble antioxidant in membranes rich in polyunsaturated phospholipids was shown by Jagannadham *et al.* [24] for Antarctic bacteria. To maintain membrane fluidity when grown at a low temperature, these bacteria synthesize a greater proportion of unsaturated fatty acids, which correlates with the synthesis of zeaxanthin.

All of the above indicates that xanthophylls (polar carotenoids) in contrast to carotenes (nonpolar carotenoids) are effectively involved in the protection of lipids in biological membranes rich in PUFAs, which are especially susceptible to oxidative destruction. Epidemiological studies indicate that a xanthophyll-rich diet and xanthophyll supplementation can impede the onset of age-related neurodegenerative diseases, such as age-related macular degeneration (AMD), Alzheimer’s disease and dementia [2,29,30,31,32,33]. The most accepted mechanism through which xanthophylls protect the neural tissue against degenerative diseases is their action as lipid-soluble antioxidants [34,35,36,37]. The direct and indirect antioxidant actions of xanthophylls involve blue light filtration [38,39], quenching of singlet oxygen [40] and scavenging of free radicals [41,42,43]. *In vitro*, these abilities of xanthophylls are not significantly better than those of other carotenoids. Therefore, it must be some specific property of these xanthophylls that could help explain their preferential (brain) or selective (retina) presence in the human neural tissue and why nature has chosen them from more than 20 other carotenoids present in blood plasma. Norman Krinsky in his reviews [44,45] indicated that one such property is the disposition of xanthophylls in biological membranes. This review is responsive to those articles with the central hypothesis that states: the high membrane solubility, preferential transmembrane orientation and selective concentration of xanthophylls in the most vulnerable regions of lipid bilayer membranes enriched in polyunsaturated lipids distinguish them from other dietary carotenoids, enhance their chemical and physical stability in retina and brain membrane and maximizes their protective action in these organs. We cannot rule out the possible function of specific xanthophyll-binding proteins that can enhance the selective uptake and deposition of macular xanthophylls in the retina and brain. Both interactions of macular xanthophylls with membranes and specific proteins are significant. However, in this review, we will discuss mainly xanthophyll-membrane interactions.

## 2. High Membrane Solubility and Transmembrane Location

Carotenoids are transported in human blood plasma exclusively by lipoproteins. The segregation of polar (xanthophylls) and nonpolar (carotenes) carotenoid molecules already occurs on the level of carotenoid transport, where carotene molecules are associated primarily with the low-density lipoproteins (LDLs) and xanthophylls are carried by the high-density lipoproteins (HDLs) [46,47]. The reported ratio of lutein in HDLs to lutein in LDLs is approximately 3:1, whereas the ratio of lycopene in HDLs to lycopene in LDLs is about 1:2 [48]. Probably, the HDL transport is critical for the delivery of xanthophylls to the neural tissue. This statement is supported by results obtained by Connor *et al.* [49], which showed the correlation between HDL deficiency and macular xanthophylls’ deposition in the chicken retina. HDL-deficient mutant chickens fed a high-lutein diet accumulated less lutein and zeaxanthin in the retinal tissue than control chickens fed the same diet. The proportion of the surface phospholipids to the core lipids (cholesteryl esters and triglycerides) in HDLs and LDLs is different. In HDLs, the ratio of phospholipids to core lipids is 1.4:1, whereas in LDLs, this ratio is 0.3:1 [48]. Furthermore, Borel *et al.* [48] have suggested that this initial segregation of carotenoids is a consequence of the preferential solubility of xanthophylls in phospholipids. HDLs are also enriched in phospholipids containing PUFAs [50]. We think that the association of xanthophylls with HDLs is a result of their high solubility in a PUFA environment. This statement is supported by the findings of Delyfer *et al.* [51], which showed that a high plasma level of total PUFAs correlates with the high concentration of xanthophylls in the retina. Additional evidence for the correlation between retinal carotenoids and lipids in HDLs has been provided by Renzi *et al.* [52].

Macular xanthophylls are very soluble in lipid bilayer membranes. The reported solubility thresholds in fluid-phase phospholipid model membranes are in the area of 10 mol% for zeaxanthin and 15 mol% for lutein [53]. A value of 5 mol% also was reported for zeaxanthin incorporated into unilamellar vesicles formed with dipalmitoylphosphatidylcholine [54]. An even lower solubility threshold in lipid bilayer membranes (about 1 mol%) was reported for canthaxanthin, a polar carotenoid with keto groups [55]. Nonpolar β-carotene starts to aggregate at a concentration as low as 0.5 mol% [56]. Mono-polar β-cryptoxanthin is less soluble in the phospholipid bilayer than dipolar xanthophylls [57]. This tendency was confirmed by Socaciu *et al.* [58], who measured the incorporation ratio of different carotenoids in different phospholipid model membranes. They confirmed high incorporation for xanthophylls and low incorporation for β-carotene. All of these indicate that the high membrane solubility of macular xanthophylls is one of the major characteristics that distinguishes them from other dietary carotenoids.

At a high concentration, xanthophylls can significantly affect membrane properties. They significantly shift the main phase transition of phospholipid bilayers to a lower temperature and decrease the cooperativity of the main phase transition [57,59]. The effect of β-carotene on the membrane phase transition is negligible. Xanthophylls also increase the order of phospholipid membranes [57] and decrease alkyl-chain motion in the fluid phase [60]. At a concentration of 10 mol%, they significantly increase the hydrophobicity of the membrane interior [61]. These effects are the strongest for dipolar xanthophylls, significantly weaker for monopolar xanthophylls (β-cryptoxanthin) and negligible for nonpolar carotenoids (β-carotene).

**Figure 3:**
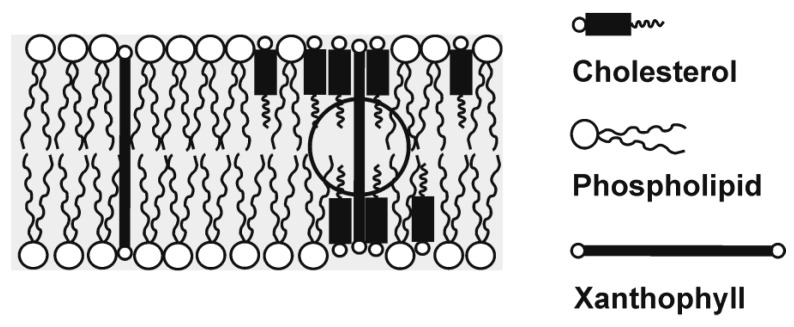
Schematic drawing showing the location of the xanthophyll molecule in the cholesterol-rich and cholesterol-poor membrane domains. An unfavorable interaction with cholesterol in the cholesterol-rich domain is indicated.

The high incorporation rate of xanthophylls into lipid membranes is reduced when cholesterol is present in the phospholipid bilayer [58,62]. The decline in incorporation was the strongest for polar carotenoid zeaxanthin. We observed a similar tendency when spin-labeled lutein was incorporated into saturated phosphatidylcholine membranes [63]. Spin-labeled lutein was completely insoluble when cholesterol (30 mol%) was present in these membranes. We think that in the phospholipid bilayer, the xanthophyll-cholesterol interaction is weaker than the xanthophyll-phospholipid interaction. Two polar groups of xanthophyll molecules interact with opposite surfaces of the membrane, and its rigid bar-like portion crosses the entire membrane. The cholesterol molecule is located in one leaflet of the bilayer, and its rigid plate-like portion extends to the depth of the seventh to ninth carbon in the lipid bilayer. When these molecules are located next to each other in the phospholipid bilayer, a free space is created in the membrane center. Cholesterol molecules are forced to sink deeper into the bilayer, which is energetically unfavorable, because it allows water to access the hydrophobic surface of alkyl chains (indicated schematically in Figure 3). These unfavorable interactions caused by xanthophylls and cholesterol are avoided by their separation in the phospholipid membranes (see Section 3).

**Figure 4:**
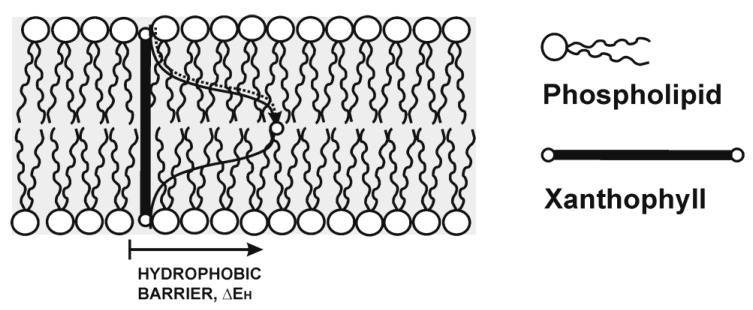
Schematic drawing explaining the physical stability of dipolar xanthophylls in the lipid-bilayer membranes. A hydrophobic barrier across the lipid bilayer is indicated. To remove the dipolar xanthophyll molecule from the bilayer, one of its polar −OH groups has to cross the hydrophobic (energy) barrier. ΔE_H_ is the energy needed to pull the polar −OH group of the dipolar xanthophyll across this barrier.

The transmembrane orientation of xanthophylls in phospholipid bilayers is well documented [64,65,66,67,68,69,70]. The presence of polar hydroxyl groups at the ends of macular xanthophyll molecules (see Figure 1) ensures their perpendicular or nearly perpendicular orientation in the bilayer (as shown in Figure 3). The transmembrane localization of macular xanthophylls in retinal membranes can also explain their very slow removal from the retina (about 50 days), observed after discontinuation of xanthophyll supplements given to healthy volunteers [71]. Similar effects were also observed by Hammond *et al.* [72]. For xanthophylls to be stable in membranes, they must anchor at opposite membrane surfaces. Cholesterol molecules, which also are anchored by the −OH group, but at only one membrane surface (located in one membrane leaflet), can be completely replaced in the retina every six to seven days, and in humans, this replacement may be even more rapid [73]. The incorporation yield of macular xanthophylls into liposomes and cells of retinal pigmented epithelium is five- to 10-times greater than the incorporation yield of canthaxanthin and 20- to 40-times greater than the incorporation yield of β-carotene [74]. These observations suggest that anchoring xanthophyll molecules at opposite membrane surfaces is significant not only in enhancing their effects on membrane properties [57,66,69], but also in stabilizing these molecules in membranes of the human retina (shown schematically in Figure 4). It should be noted that Sujak *et al.* [75] reported results indicating the existence of two orthogonally-oriented pools of lutein in model phosphatidylcholine membranes, one following the orientation of zeaxanthin (perpendicular to the membrane surface) and the second parallel to the membrane surface. Furthermore, Monte Carlo [76] and molecular dynamics [77] simulations of the behavior of lutein in phosphatidylcholine bilayers have shown that these two orientations are very probable. The existence of two orthogonally-oriented pools of lutein in the membrane may enhance its physiological role as a blue light filter. All of the above allowed us to conclude that solubility, orientation and organization of macular xanthophylls in lipid bilayer membranes depend on their structure, as well as on the lipid environment.

Interesting conclusions can be made by comparing the antioxidant properties of xanthophylls to the antioxidant properties of other dietary carotenoids investigated in organic solvents and in lipid bilayer membranes. Zeaxanthin and β-carotene show similar antioxidant properties in organic solutions. However, their antioxidant properties differ when incorporated into membranes [78]. Zeaxanthin was shown to react with free radicals slightly more effectively than β-cryptoxanthin and much more effectively than β-carotene [79,80]. β-carotene and lycopene are able to react efficiently only with radicals generated inside the membrane. Xanthophylls, with their hydroxyl groups exposed to an aqueous environment, can also scavenge free radicals generated in the aqueous phase [81]. The above examples allow us to conclude that the presence of polar hydroxyl groups at the ends of xanthophyll molecules and their transmembrane orientation enhance their stability in membranes and their antioxidant properties and, thus, maximize their protective action in biological membranes. Those properties also distinguish xanthophylls from other dietary carotenoids.

## 3. Location in the Most Vulnerable Regions of Lipid Membranes

In the human retina, the concentration of xanthophylls reaches a level between 0.1 and 1 mM in the central fovea [5,71]. Xanthophylls are accumulated mainly in the region of photoreceptor axons [5], where they act as a blue light filter, and within photoreceptor outer segments (POS) [6,7], where their antioxidant functions are assumed. Although xanthophylls in POS constitute about 10% to 25% of the amount in the entire retina [6,7], the local concentration of xanthophylls in membranes of the rod outer segment is ~70% higher than in residual retina membranes [7]. The concentration of xanthophylls in the human retina is, however, high enough for effective blue light filtration and antioxidant action. To understand the basic mechanisms of these actions, it is necessary to better understand the carotenoid-membrane interaction. For systems with a high carotenoid concentration (in bacteria and plants where the local carotenoid concentration in the lipid bilayer can reach a value of a few mol%), it is most significant to understand how carotenoids affect membrane physical properties, structure and dynamics, as well as the lateral organization of the lipid bilayer (its domain structure). For systems with a low carotenoid concentration, it is especially important to understand how the membrane itself—membrane composition, structure and lateral organization—affects the organization of carotenoids in the lipid bilayer, including their solubility (monomeric *versus* aggregated state), orientation (transmembrane *versus* parallel) and localization (distribution between membrane domains). Furthermore, knowledge of the bulk-membrane physical properties, which are not uniform across the lipid bilayer and can differ by membrane domain, is significant to better understand chemical reactions and physical processes that take place in the lipid bilayer membrane and involve carotenoids [82].

Retinal pigment epithelium membranes and disk membranes of rod outer segments are laterally heterogeneous and contain raft domains [83,84,85,86]. Raft domains, enriched in cholesterol and sphingolipids, were isolated as detergent-resistant membrane (DRM) fractions. Remaining detergent-soluble membrane (DSM) fractions, formed by bulk lipids surrounding raft domains, were rich in long-chain PUFAs. Additionally, rhodopsin, the main protein of POS membranes that is responsible for the first stages of visual signal transduction and is located in the bulk domain of the POS membrane [87], was isolated mostly with the DSM fraction [83,84,86]. Rafts in photoreceptor cell membranes are involved in the regulation of the G-protein-mediated pathway of phototransduction [85]. Aggregation of small, unstable rafts in bigger platforms (observed, for example, in retinal pigment epithelium cells) is supposed to enhance signal transduction to the cell interior and cause a specific reaction in the cell, such as apoptosis [88].

Rhodopsin requires the presence of polyunsaturated lipids (DHA) for its activity [89,90,91], and thus, their colocalization is functionally justified. Furthermore, phospholipids containing very long-chain PUFAs (VLC-PUFAs, >C24, with 3 to 9 double bonds) likely play a unique and important role in the retina because they are necessary for cell survival, and their loss leads to cell death [92,93]. It has been suggested that they are tightly bound to rhodopsin and that their unusually long chains may partially surround the α-helical segments of rhodopsin [94]. Epidemiological studies of long-chain polyunsaturated phospholipid intake suggest a protective role against the incidence of advanced AMD [95,96]. It was shown that lipids containing VLC-PUFAs are reduced in the retina of aged eyes and severely reduced in the retina of AMD eyes [97]. As indicated by the authors, their results support the potential value of interventions to increase retinal VLC-PUFAs in the prevention and treatment of AMD. All of these indicate that the colocalization of rhodopsin with polyunsaturated lipids is functionally justified. However, this colocalization creates a dangerous situation for both rhodopsin and polyunsaturated lipids, especially during illumination, when reactive oxygen species can be produced by photosensitizers [98,99]. It should be noted that phospholipids containing long-chain and very long-chain PUFAs, which are involved in preventing AMD, are also very prone to oxidative damage. Fortunately, to protect the retina against oxidative damage, nature has used xanthophylls as an effective protector that can absorb damaging blue light, neutralize photosensitizers and reactive oxygen species and scavenge free radicals.

**Figure 5:**
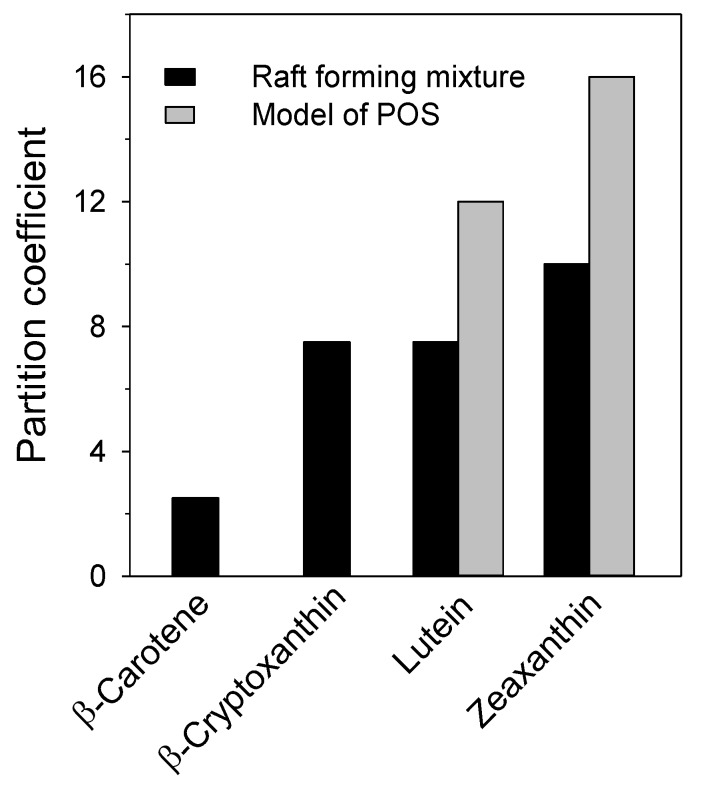
Partition coefficient of dipolar xanthophylls (lutein and zeaxanthin), monopolar xanthophyll (β-cryptoxanthin) and nonpolar carotenoid (β-carotene) between the bulk (unsaturated) domain and the raft (saturated) domain in the membrane made of the raft-forming mixture and in the model of photoreceptor outer segment (POS) membranes. The unsaturated domain in the model of POS membranes is abundant in DHA, with six double bonds. For more details, see [100,101].

Our investigations, made on a model of POS membranes, indicate that xanthophylls were about 14-times more concentrated in the unsaturated bulk domain (enriched in polyunsaturated lipids and isolated as DSM) and excluded from the raft domain (enriched in saturated lipids and cholesterol and isolated as DRM) [100]. A similar distribution also was found in membranes made of a raft-forming mixture where macular xanthophylls lutein and zeaxanthin were about eight-times more concentrated in the bulk, unsaturated domain than in the raft domain [101]. A similar distribution has been observed for monopolar β-cryptoxanthin, but not for nonpolar β-carotene, which was more uniformly distributed between the DRM and DSM domains. Results indicating the distribution of carotenoids between raft and bulk domains are summarized in Figure 5. Colocalization of xanthophyll molecules with polyunsaturated lipids is ideal if xanthophylls are to act as a lipid antioxidant, which is the most accepted mechanism through which lutein and zeaxanthin protect the retina from AMD [44,45,102]. It should significantly enhance the effectiveness of xanthophylls, especially when the local concentration of xanthophylls in the membrane is not very high. Studies by Wisniewska-Becker *et al.* [103] show that preferential colocalization of xanthophylls with polyunsaturated lipids really enhances their antioxidant activity. The observed inhibition of lipid peroxidation by lutein was significantly greater in membranes containing raft domains than in homogenous membranes.

## 4. Photo-Related and Dark Antioxidant Action

**Figure 6:**
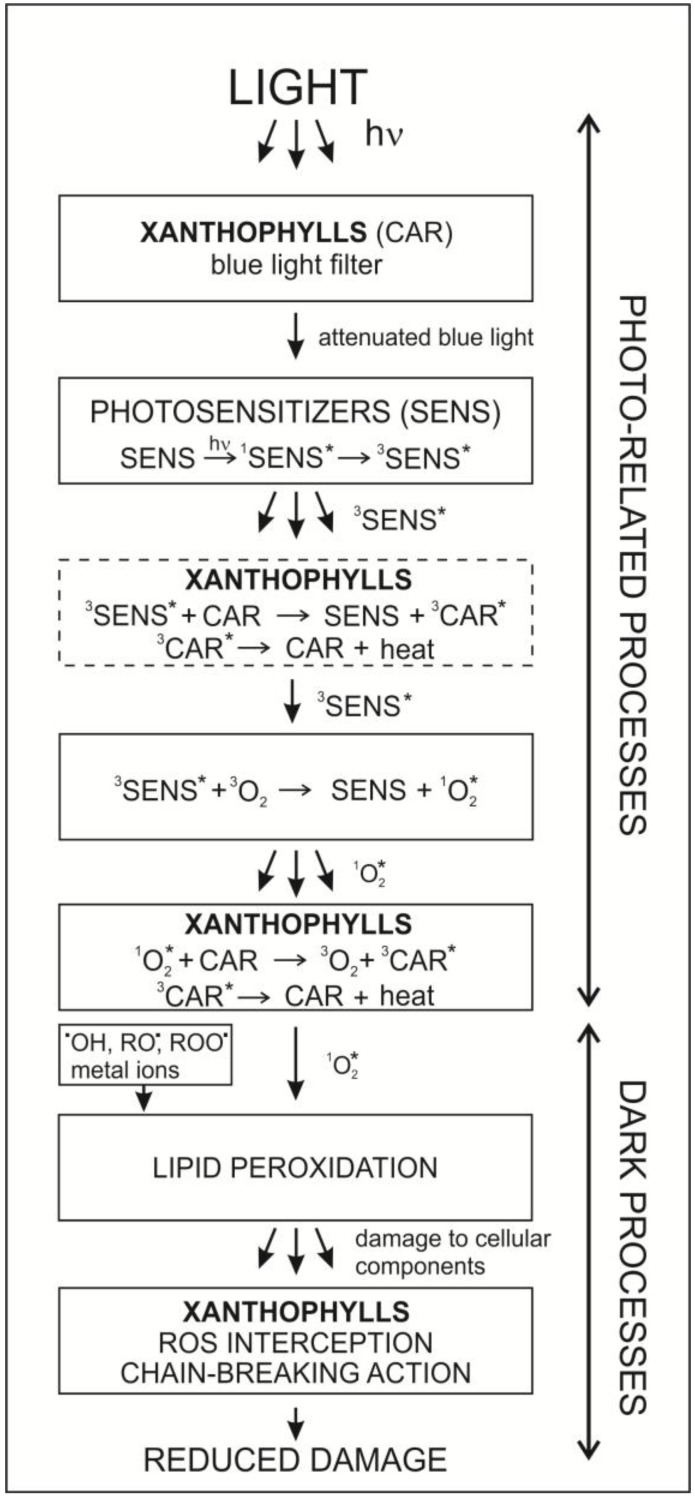
Diagram illustrating processes through which xanthophylls are protecting membranes against oxidative damage. Photo-related and dark processes are actively involved in protecting the retina, while only dark processes are assumed to be active in the protection of brain tissue. The broken rectangles indicate processes that are not yet fully confirmed as involved in protecting the eye retina and are included here as purported processes.

It is commonly believed that xanthophylls protect eye retinas mainly through photo-related antioxidant action, while in the brain, the dark antioxidant action of xanthophylls is expected. In this section, we will describe the mechanisms of both actions, which are summarized in the diagram in Figure 6.

In the retina, macular xanthophylls are localized mostly in Henle’s layer [64] and form a filter for blue light. Although most ultraviolet is absorbed by the cornea [104] and lens [105], some fraction of blue radiation reaches the retina, may activate potent retinal photosensitizers (such as all-trans retinal, cytochrome c oxidase, porphyrins and the major chromophore of lipofuscin, bisretinoid A2E [99,106,107,108,109]) and, consequently, generates reactive oxygen species. Thus, blue light absorption can be considered an indirect antioxidant action, because it prevents blue light from generating reactive oxygen species that can damage photoreceptor cells. Blue light absorption by macular xanthophylls is extremely important for young eyes, for which lens transparency is almost 95%. During aging, the lens gradually loses its transparency, becomes yellowish [105] and better filters UV and blue light. Thus, in older age, the blue light filtration performed by macular xanthophylls becomes relatively less important.

The blue light filtration antioxidant mechanism is based on the high extinction coefficient of macular xanthophylls for the absorption spectrum band of 390 to 540 nm with maximum absorption at around 450 nm. The most damaging impact on the retina has been shown for exposure to high-energy light with maximum absorption at around 440 nm [99,110], which is also maximum absorption for xanthophyll molecules. Xanthophylls dissolved in lipid bilayers may exist not only in the form of monomers, but also in aggregate arrangements. Two formed aggregates are well known: the “card-pack” arrangement (H-aggregate), where the shift to a shorter wavelength is observed (blue shift), and the “head-to-tail” organization (J-aggregate), with the shift to a longer wavelength (red shift). Typically, in phospholipid membranes, macular xanthophylls are present as monomers or can form H-aggregates with a blue-shifted absorption spectrum and maximum absorption at 380 nm. Sujak *et al.* [54] observed that, in lipid bilayers, lutein has a higher degree of aggregation than zeaxanthin and, because of that, possesses a wider bandwidth in UV and blue light absorption. Interestingly, infant retinas, which have more exposure to blue and ultraviolet light, have more lutein and less zeaxanthin relative to adult retinas [17]. Better filtration of blue light by lutein may justify its unique distribution in the adult retina and its high concentration in the infant retina. Junghans *et al.* [111] have investigated the blue light filter efficiency of four plasma carotenoids (lutein, zeaxanthin, β-carotene and lycopene) incorporated into membranes of liposomes loaded with a hydrophilic fluorescent dye, Lucifer yellow, excitable by blue light. Fluorescent emission of the dye was lower in liposomes with carotenoids as compared to the control, indicating a filter effect. Macular xanthophylls lutein and zeaxanthin exhibited the highest blue light absorption activity as compared to liposomes containing nonpolar carotenoids. Blue-light filter efficacy is ordered as lutein > zeaxanthin > β-carotene > lycopene. These results indicate that xanthophylls (especially lutein) are the best blue light filters among all carotenoids available in blood plasma.

In the retina, macular xanthophylls may not only act as blue light filters, but may also optimize visual performance. The layer of macular xanthophylls is believed to reduce chromatic aberrations, glare disability and light scattering, which enhance vision contrast [112,113]. This way, xanthophylls can positively affect the cognitive function of the brain, especially in the aged population [114,115,116].

Xanthophylls are capable of quenching excited triplet states of potent singlet oxygen photosensitizers. These quenching processes belong to the direct photo-related antioxidant actions of xanthophylls. By this mechanism, the largest part of excess energy is transferred from potentially harmful triplets of photosensitizers to xanthophylls and dissipated as heat. This mechanism is well established for porphyrins, but has not yet been clearly confirmed to work for photosensitizers in the retina. In the retina, photo activation of rhodopsin leads to isomerization of its chromophore, 11-*cis*-retinal to all-*trans*-retinal, which, under certain conditions, can act as a photosensitizer. Free all-*trans*-retinal may absorb light and transfer energy from its excited triplet state to molecular oxygen, generating singlet oxygen [98]. It is postulated that the close proximity of xanthophylls, which are also located in the bulk domain (see Section 3), allows effective energy transfer from excited all-*trans* retinal to xanthophyll and prevents singlet oxygen generation by this photosensitizer [117].

Carotenoids have been known to be the most effective singlet oxygen quenchers, and their activities are much higher than that of other retinal antioxidants, tocopherols and thiols [118,119]. They are able to quench singlet oxygen by two different mechanisms. The first mechanism, which involves energy transfer, is termed physical quenching and is considered the major pathway of singlet oxygen deactivation. According to this mechanism, carotenoid molecules deactivate singlet oxygen to the nonreactive triplet state. During that process, carotenoid molecules become excited to the triplet state and can return to the ground state, dissipating the excess energy as heat. The profit of the physical quenching is that carotenoids may act without alternation of their own chemical structure. The second mechanism is called chemical quenching. It involves a chemical reaction between carotenoid and singlet oxygen, which results in pigment autoxidation. The capacity of major plasma carotenoids to quench singlet oxygen in an organic solvent mainly depends on the number of conjugated double bonds in the chromophore, but also varies with functional groups [120]. Thus, zeaxanthin (with its 11 conjugated double bonds) has a higher ability to quench singlet oxygen than lutein (with its 10 conjugated double bonds) (see Figure 1). The inactivation of singlet oxygen may also occur through chemical quenching involving autoxidation of the carotenoids, a process that consumes the carotenoids themselves. Chemical quenching is reported to contribute less than 0.05% to the overall singlet oxygen quenching by carotenoids [121]. Nevertheless, it has been reported that the retina contains macular xanthophyll metabolites, indicating that chemical quenching can take place in biological tissues [122,123]. The degradation of four major plasma carotenoids, induced by UV light in the presence of rose bengal, has been studied by van Kuijk and his co-workers [124,125]. Higher degradation rates were found for nonpolar carotenoids as compared to macular xanthophylls. Furthermore, studies of the autoxidation of carotenoids incorporated in pig liver microsomes [126] give similar results: nonpolar carotenoids, such as β-carotene and lycopene, had degraded totally, whereas polar carotenoids had degraded much slower, and zeaxanthin was shown to be the most stable carotenoid. We can conclude that the high chemical stability of macular xanthophylls distinguishes them from other dietary carotenoids.

The conjugated double bond system is primarily responsible for the high chemical reactivity of carotenoids, not only with singlet oxygen [118,120], but also with free radicals [127]. Carotenoids are capable of intercepting peroxyl radical and inhibiting phospholipid peroxidation. Selective localization of xanthophylls in domains rich in polyunsaturated phospholipids (see Section 3) and, therefore, susceptible to free-radical-induced damage is ideal for their chemical dark antioxidant action. Carotenoids scavenge lipid peroxyl radicals by forming radical adducts [127] that are less reactive than lipid alkyl peroxyl radicals. Thus, carotenoids are effective chain-breaking antioxidants, which delay the oxidation of biological membranes by trapping the chain-initiating or chain-propagating peroxyl radicals. This dark antioxidant action should form the major mechanism through which xanthophylls protect brain tissue against neurodegenerative diseases. However, direct mechanisms of xanthophyll actions in the brain are not yet investigated in detail and not yet defined.

## 5. Concluding Remarks

Carotenoids are important dietary prophylactic agents in numerous degenerative diseases. Among them, macular xanthophylls play a unique position in their influence on the health and functions of the retina and brain. Their selective presence throughout the central nervous system tissue has led to the questions of not only how these dietary carotenoids influence the retina and brain, but also why nature, during the evolutionary process, chose macular xanthophylls from other carotenoids available in the human diet to perform those specific functions. In this review, we informed researchers about the importance of the xanthophyll-membrane interactions that enhance their chemical and physical stability in the retina and brain membranes and maximize their protective action in these organs.

In this section, we make additional comments about the selective accumulation of macular xanthophylls, lutein and zeaxanthin, in the outer plexiform layer (which comprises a dense network of synapses in the retina) [64]. It is commonly accepted that the primary function for these xanthophylls is to provide a filter for blue light [111]. We think that equally significant is the direct antioxidant action of xanthophylls to protect membranes of neuronal endings and synaptosomes, which are rich in polyunsaturated phospholipids. In a similar manner, xanthophylls can act in the phospholipid environment of the grey matter of the brain, which is rich in PUFAs [128,129]. Interestingly, in these two neural tissues, only polar carotenoids (namely xanthophylls) are present as lipid-soluble antioxidants. Embryologically, the retina is part of the brain. Additionally, polyunsaturated phospholipids, especially DHA, are present in high amounts in the retina (rod outer segment and synaptic area) and in the brain, primarily in synaptosomes [93,130,131,132]. Retina and brain tissues are also very well oxygenated, which makes them particularly accessible to oxidative damage. Thus, they need relatively stable lipid-soluble antioxidants for their protection. Data indicate that xanthophylls serve this function well.

Throughout this review, we indicated that there are several physical and chemical properties that distinguish xanthophylls, especially macular xanthophylls, from other carotenoids available in the human diet as lipid-soluble antioxidants of neural tissues. They include high membrane solubility, preferential transmembrane orientation and selective concentration in the polyunsaturated lipid environment. Xanthophylls also have greater resistance to autoxidation than carotenes, which is an important feature, especially for neural tissue, where turnover of the antioxidant pole is slow. All of these unique properties enhance xanthophylls’ chemical and physical stability in biological membranes and maximize their protective actions. Less chemically-stable carotenes can be more easily oxidized in lipid bilayer membranes [126] and can even function as prooxidant compounds [133,134]. These unique properties were “recognized” during evolution when mechanisms for the selective accumulation of xanthophylls in the retina and brain were developed. These mechanisms are not yet understood in detail. Some suggestions about selective xanthophyll transport are included in Section 2. Selective accumulation suggests involvement of specific xanthophyll-binding proteins. Some of these membrane-associated, xanthophyll-binding proteins already have been identified and characterized, including zeaxanthin-binding protein [135] and lutein-binding protein [136]. The unanswered question is whether these proteins are only selective transporters of macular xanthophylls or whether they can store xanthophylls.

It is clear from Figure 2A that evolution selected xanthophylls, including lutein and zeaxanthin, to play their protective role in neural tissue. However, we found very intriguing the fact that the human diet is poor in zeaxanthin and relatively rich in lutein, while comparatively, the amount of zeaxanthin in the retina and brain is significantly enhanced (see Figure 2). Zeaxanthin, with its 11 double conjugated bonds, has a higher ability to quench singlet oxygen than lutein, with its 10 conjugated double bonds [120] (see Figure 1). This property of zeaxanthin as the best lipid-soluble antioxidant among xanthophylls was recognized during evolution. We also think that the evolutionary selection of zeaxanthin as the best lipid-soluble antioxidant in different organisms (bacteria, plants, humans) and tissues (human retina, human brain) is associated with the requirement to protect the highly unsaturated regions of the lipid membranes. To compensate for the poor presence of zeaxanthin in the human diet, nature developed a mechanism to transform lutein, which is the most abundant xanthophyll in the diet, to an isomer of zeaxanthin, namely *meso*-zeaxanthin, directly in the retina (see Figure 1 for the chemical structures). The detailed mechanism of this isomerization process is unknown; however, we think that the nature of the overall strategy (appearance of zeaxanthin molecules in phospholipid environment susceptible to oxidation) used here is similar to that in plants during the violaxanthin cycle [137]. During this cycle, violaxanthin is partially transformed to zeaxanthin through chemical de-epoxidation, whereas during isomerization of lutein in the retina, rearrangement of bonds within the functional groups occurs [17,138]. We can summarize that the distribution of xanthophylls in human tissues and organs is biologically controlled by their solubility, specific binding proteins (this control is structure dependent, distinguishing zeaxanthin and lutein) and the site of formation (as in the case of *meso*-zeaxanthin).

The neuronal conduction and synaptic neurotransmission is affected by the membrane state. Neuronal lipid bilayer membranes in the brain and retina are especially vulnerable to oxidative damage because of their enrichment in PUFAs and exposure to high oxygen concentration. Only dipolar xanthophylls, with their high solubility in polyunsaturated phospholipid membrane domains and high resistance to autoxidation, serve the role of protector in these neural membranes. Lutein and zeaxanthin are present at a high concentration in the synaptosomal membranes of the outer plexiform layer, which links the brain and retina tissues. Although the most accepted function of these xanthophylls is blue light filtration, we postulate that equally significant is the protection of these highly unsaturated membranes through the trapping of chain-initiating and/or chain-propagating peroxyl radicals. Through this function, xanthophylls can effectively protect the neuronal synapses region in both the retina and brain tissues. This dark antioxidant action is indicated in Figure 6.
